# Survival advantages conferred to colon cancer cells by E-selectin-induced activation of the PI3K-NFκB survival axis downstream of Death receptor-3

**DOI:** 10.1186/1471-2407-11-285

**Published:** 2011-07-01

**Authors:** Nicolas Porquet, Andrée Poirier, François Houle, Anne-Laure Pin, Stéphanie Gout, Pierre-Luc Tremblay, Éric R Paquet, Roscoe Klinck, François A Auger, Jacques Huot

**Affiliations:** 1 Le Centre de recherche en cancérologie de l'Université Laval et Centre de recherche du CHUQ, l'Hôtel-Dieu de Québec, 9 rue McMahon, Québec G1R 2J6 Canada; 2 Institute of Developmental Biology and Cancer, CNRS UMR6543, Université Nice Sophia Antipolis 06108 Nice Cedex 2, France; 3 Centre de recherche Inserm/UJF U823 Equipe 2, Institut Albert Bonniot BP 170, 38052 Grenoble Cedex 09, France; 4 Laboratoire d'Organogenèse Expérimentale, Centre hospitalier affilié Universitaire de Québec, 1401, 18erue Québec, G1J 1Z4, Canada; 5 Laboratoire de Génomique Fonctionnelle de l'Université de Sherbrooke, 3201, rue Jean Mignault, Sherbrooke J1E 4K8 Canada

**Keywords:** Death receptor-3, E-selectin, colon cancer, PI3 kinase, splice variant

## Abstract

**Background:**

Extravasation of circulating cancer cells is a key event of metastatic dissemination that is initiated by the adhesion of cancer cells to endothelial cells. It requires interactions between adhesion receptors on endothelial cells and their counter-receptors on cancer cells. Notably, E-selectin, a major endothelial adhesion receptor, interacts with Death receptor-3 present on metastatic colon carcinoma cells. This interaction confers metastatic properties to colon cancer cells by promoting the adhesion of cancer cells to endothelial cells and triggering the activation of the pro-migratory p38 and pro-survival ERK pathways in the cancer cells. In the present study, we investigated further the mechanisms by which the E-selectin-activated pathways downstream of DR3 confer a survival advantage to colon cancer cells.

**Methods:**

Cell survival has been ascertained by using the WST-1 assay and by evaluating the activation of the PI3 kinase/NFκB survival axis. Apoptosis has been assayed by determining DNA fragmentation by Hoechst staining and by measuring cleavage of caspases-8 and -3. DR3 isoforms have been identified by PCR. For more precise quantification, targeted PCR reactions were carried out, and the amplified products were analyzed by automated chip-based microcapillary electrophoresis on an Agilent 2100 Bioanalyzer instrument.

**Results:**

Interaction between DR3-expressing HT29 colon carcinoma cells and E-selectin induces the activation of the PI3K/Akt pathway. Moreover, p65/RelA, the anti-apoptotic subunit of NFκB, is rapidly translocated to the nucleus in response to E-selectin. This translocation is impaired by the PI3K inhibitor LY294002. Furthermore, inhibition of the PI3K/Akt pathway increases the cleavage of caspase 8 in colon cancer cells treated with E-selectin and this effect is still further increased when both ERK and PI3K pathways are concomitantly inhibited. Intriguingly, metastatic colon cancer cell lines such as HT29 and SW620 express higher levels of a splice variant of DR3 that has no trans-membrane domain and no death domain.

**Conclusion:**

Colon cancer cells acquire an increased capacity to survive via the activation of the PI3K/NFκB pathway following the stimulation of DR3 by E-selectin. Generation of a DR3 splice variant devoid of death domain can further contribute to protect against apoptosis.

## Background

The metastatic process consists of a number of sequential interrelated steps, all of which must be completed successfully to give rise to a secondary tumor [1-3]. In particular, the adhesion of cancer cells to endothelial cells is a prerequisite for extravasation of circulating cancer cells and for their metastatic dissemination. This adhesive event requires specific interactions between adhesion receptors present on vascular endothelial cells and their ligands or counter-receptors on cancer cells. E-selectin is a specific endothelial adhesion receptor that is induced by pro-inflammatory stimuli. Its natural function is to mediate the adhesion of leukocytes to the endothelium allowing their extravasation into inflamed tissues [4]. Intriguingly, cancer cells hijack the inflammatory system and interact with E-selectin to extravasate [5,6]. For example, colon carcinoma cells adhere to and roll on both purified E-selectin and cytokine-stimulated endothelial cells either in static or dynamic conditions *in vitro *[7-9]. Moreover, several studies strongly support the role of E-selectin-mediated adhesion of cancer cells to endothelial cells as an important determinant of metastasis, especially of colon carcinoma cells. In particular, the binding efficiency of clonal colon cancer cell lines to E-selectin is directly proportional to their respective metastatic potential [10]. In contrast, anti-E-selectin antibodies and antisense oligonucleotides that inhibit E-selectin expression impair experimental liver metastasis of murine and human tumor cells [11,12]. Similarly inhibiting the expression of E-selectin with cimetidine, an antagonist of histamine H2 receptors, inhibits the adhesion of cancer to endothelial cells and impairs metastatic dissemination [13].

The binding of cancer cells to E-selectin involves a counter-receptor for E-selectin that is composed of sialyl Lewis-a/x carbohydrate determinants that are borne by a carrier protein or lipids on cancer cells. The binding is Ca^2+^-dependent and is mediated through the N-terminal lectin domain of E-selectin. Sialyl Lewis-a on carrier proteins plays a major role in E-selectin binding of cancer cells derived from the lower digestive organs, such as the colon and rectum, as well as from the pancreas and biliary tract [14]. On the other hand, sialyl Lewis-x is the representative carbohydrate involved in the E-selectin binding of breast, ovarian and pulmonary cancer cells [1]. Little is known about the proteins that bear these carbohydrates and that serve as the E-selectin counter-receptor backbone on cancer cells. LAMP-1, LAMP-2, CD44, CEA and podocalyxin-like proteins were all identified as E-selectin counter-receptors on colon cancer cells [15-19]. However, the signaling events that stem from these receptors in the cancer cells bound to E-selectin are still ill defined. Several studies have shown that the adhesion of cancer cells to E-selectin initiates a reverse signaling in the cancer cells, which raises the possibility that this signaling modulates the metastatic potential of cancer cells [20-22]. We previously reported that Death receptor-3 (DR3) is a functional and signaling sialylated ligand that binds E-selectin on colon cancer cells [20,23]. The subsequent DR3 activation induced by E-selectin increases the motile potentials of the cancer cells through activation of the p38 MAP kinase pathway [20,23].

DR3 is a member of the second group of the TNF receptor (TNFR) superfamily that includes TNFR1, DR4, DR5, DR6, and Fas [17]. These receptors contain a common 70- to 80-amino acid homologous region in the cytoplasmic tail called the death domain [24]. The signaling pathways leading to cell death in response to these receptors are similar and rely on trimerization and oligomerization of the receptors upon ligand binding followed by the recruitment of death domain proteins, such as TRADD, FAD, or RIP1, and subsequently, activation of the apoptotic cascade [25]. More recently, it was reported that CD95/Fas, a member of the TNFR family, induces signaling to phosphatidylinositol 3-kinase (PI3K) via phosphorylation of Tyr residues present in its death domain [26]. Several splice isoforms of DR3 exists, some of which such as, isoforms 1, 2, 3, 4 and 7, contain a death domain, while others, such as the truncated DR3 isoform 12, do not [27]. Among these variants, DR3 isoform 2 (DR3v2) is the major and parental member of the family and is referred to hereafter as DR3. Interestingly, the splicing profile of DR3 may be altered in cancer. Notably, DR3β differs from DR3 by the inclusion of a 28 amino-acid stretch in the extracellular domain. Whereas DR3 is expressed in all cell lines and lymphoma samples tested, DR3β expression is restricted to lymphoid T-cell and immature B-cell lines and to some cases of follicular lymphoma. This suggests that several receptor isoforms can participate in lymphoid cell homeostasis [28]. The functions of DR3 in a physiopathologic context are unclear. However, its ectopic expression in mammalian cells induces apoptosis or activates the pro-survival transcription factor NFκB, depending on the cytoplasmic effectors engaged in the signaling complexes downstream of the death domain [29,30]. Intriguingly, the activation of DR3 by TL1A/VEGI, the cognate ligand for DR3 is not followed by apoptosis in human erythroleukemic TF-1 cells. This is presumably because it is associated with the expression of the apoptosis-inhibiting protein c-IAP2 [31,32]. More recently, we found that activation of DR3 by E-selectin increased the survival of LoVo colon cancer cells, in part by activating the ERK pathway [23].

In this study, we further investigated the mechanisms by which activation of DR3 by E-selectin increases the survival of colon carcinoma cells. Our major finding is that metastatic colon cancer cells do not enter into apoptosis in response to E-selectin in part because they bind to DR3 to activate the PI3K/NFκB survival pathway and in part because they generate an alternative splice variant of DR3 that lacks trans-membrane and death domains, thus rendering it unable to induce apoptosis.

## Methods

### Reagents and antibodies

Recombinant human E-selectin/Fc (rhE-selectin/Fc) was obtained from R&D Systems (Minneapolis, MN). Phenylethylisothiocyanate (PEITC) and LY294002 were purchased from Sigma (St Louis, MO). Calcein-AM was obtained from Invitrogen-Molecular Probes (Burlington, ON, Canada). Dimethylsulfoxyde was purchased from Fisher (Montreal, QC, Canada). Protein G-sepharose was purchased from GE Healthcare (Mississauga, ON, Canada). PP2 and PD098059 were purchased from Calbiochem (Mississauga, ON, Canada). Rabbit anti-DR3 clone H300 was obtained from Santa Cruz biotechnology, mouse anti-DR3 extracellular domain (DR3ecd), mouse anti-vinculin (hVIN-1), rabbit anti-active caspase 3, and irrelevant mouse IgG1κ (MOPC21) were purchased from Sigma (St Louis, MO). Mouse anti-DR3 clone B65 was obtained from Millipore (Nepean ON, Canada). Mouse anti-DR3 (hDR3) was purchased from R&D Systems (Minneapolis, MN). Rabbit anti-phospho Akt (Ser 473), rabbit anti-Akt, rabbit anti-NFκB p65 and mouse anti-caspase 8 (1C12) were all obtained from Cell Signaling Technology, (Beverly, MA). Mouse anti-TATA Binding Protein (TBP) antibody was purchased from AbCam (Cambridge, MA). Goat anti-mouse IgG (H+L) and goat anti-rabbit IgG (H+L) conjugated with horseradish peroxidase were from Jackson Immunoresearch (West Grove, PA).

### Cells

HT29 colorectal adenocarcinoma cells were cultivated in McCoy 5A medium supplemented with 10% foetal bovine serum (FBS) and antibiotics. HT29LMM (highly metastatic HT29 cells) and Jurkat T cells were cultivated in RPMI medium containing 10% FBS. Caco2 colorectal adenocarcinoma cells were grown in DMEM high glucose medium supplemented with 10% FBS and Glutamax 1X. SW480 and SW620 are colorectal adenocarcinoma cells isolated from the primary site and lymph node secondary site from the same patient. They were cultivated in Leibovitz medium L15 containing 10% FBS. LoVo colorectal adenocarcinoma cells grade IV were grown in Ham F12K medium supplemented with 10% FBS. HIEC cells are normal human intestinal epithelial cells that were cultivated in OptiMEM containing 5% FBS and 5 ng/ml EGF [33]. HEK293, HeLa, MDA MB231 and MCF7 cells were cultivated in DMEM containing 10% foetal calf serum. All these cell lines were obtained from ATCC.

Human umbilical vein endothelial cells (HUVEC) were isolated by collagenase digestion of umbilical veins from undamaged sections of fresh cords, as described [34]. The cells used at passages ≤ 5 were grown to confluence in gelatin-coated tissue culture flasks in medium 199 containing 20% heat-inactivated FBS, endothelial cell growth supplement (60 μg/ml), glutamine (2 mM), heparin (25000 IU). Human micro-capillary endothelial cells (HMEC) were cultivated in MCDB medium containing 10% FBS, 1 μg/ml hydrocortisone and 10 ng/ml EGF. All cells lines were cultivated in the presence of antibiotics and maintained at 37°C in a 5% CO_2 _humidified atmosphere.

### Adhesion assays in a laminar flow chamber

HUVEC were trypsinized and grown for 24 hrs on gelatin-coated slides. These endothelial cells were treated with 20 ng/ml IL-1β for 4 h to induce the expression of E-selectin. The cultures were then placed in the laminar flow chamber GlycoTech (Gaithersburg, MD, USA) under a shear stress of 1 dyne/cm^2^. In certain experiments, anti-human DR3 monoclonal Ab clone B65 or MOPC21 irrelevant antibody were added in the culture medium of HT29 cells, 30 min before their injection in the chamber. In other experiments, a knockdown of DR3 was performed by small interfering RNA, as previously described [9,23]. Briefly, HT29 cells were transfected by electroporation with human DR3 siRNA (siRNA; sense, 5'-CCGUCCAGUUGGUGGGUAA-3', and antisense, 5'-UUACCCACCAACUGGACGG-3') or control siRNA purchased from Qiagen (Mississauga, ON, Canada). Tumor cells in suspension (2 × 10^6 ^per assay) were labeled for 30 min with Calcein AM and washed twice with M199 medium before being added into the flow chamber. Videos were taken directly using a camera mounted on a TE2000 fluorescence microscope at ×20 magnification (Nikon, Melville, NY, USA).

### Survival assay

Twenty-four hours after being plated, HT29 cells were left to grow for 96 hours with or without E-selectin or with the apoptosis inducer curcumin (75 μM) [35]. At the end of the treatments, the cell survival was evaluated with the Quick Cell Proliferation Assay Kit from BioVision (Mountain View, CA). The test evaluates the ability of viable cells to convert tetrazolium salt into formazan (WST-1 assay), which can be monitored at 450 nm.

### PI3 kinase and NFκB activation

Cells were washed twice and incubated in serum-free medium for 2 hours in the presence or not of the inhibitors (LY294002 or PP2). Thereafter, rhE-selectin was added for different periods of time. Cell extracts were prepared and PI3K and NFκB activation were assayed in western blotting by determining the phosphorylation of Akt at Ser 473 and nuclear translocation of p65NFκB, respectively.

### Extraction of nuclear proteins in denaturing conditions

The protocol was adapted from Andrews and Faller [36]. Cells were washed 3 times in PBS and were re-suspended in 1.6 ml of PBS. The cell suspension was briefly vortexed and 100 μl of total extract were collected and mixed in 20 μl of extraction buffer. The rest of the cell suspension was centrifuged (16,000 × g) for 10 seconds at 4°C, and the pellet was resuspended in 400 μl of buffer A (10 mM HEPES-NaOH pH 7.9, 1.5 mM MgCl_2_, 10 mM KCl, 0.5 mM DDT, 0.2 mM PMSF). The extract was left on ice for 10 min, vortexed for 10 seconds and centrifuged for 10 seconds at 4°C. The supernatant was removed and discarded, and the pellet was resuspended in 70 μl of buffer C (20 mM HEPES-NaOH pH 7.9, 25% glycerol, 420 mM NaCl, 1.5 mM MgCl_2_, 0.2 mM EDTA, 0.5 mM DDT, 0.2 mM PMSF). The samples were incubated on ice for 20 minutes and centrifuged for 2 min at 4°C. Extraction buffer was added in each extract before heating. The amount of proteins was quantified by the Lowry method.

### DR3 sequencing

Total RNA was extracted from (1 × 10^6^) cells using Qiagen RNeasy kit (Mississauga, ON, Canada). All RNA samples were stored at -80°C until assay. The mRNA was reverse-transcribed with Qiagen Sensiscript reverse transcription kit using random hexamers. Nested PCRs were used to amplify a fragment of the *tnfrsf25 *gene using specific pairs of primers and the Qiagen Hotstart taq DNA polymerase kit according to the manufacturer protocol (PCR1-Forward primers: 5'-CGTCGGAGGGCTATGGAGCAGC-Reverse primers: 5'-GGCCGGCTGGTGCTGCTACGC. PCR2-Forward primers: 5'-GAGGATCCATGCAGGGCGGCACTCGTAGC-Reverse primers: 5'-ACCTCGAGTCACGGGCCGCGCTGCAG). PCR products were cloned in pcDNA3 (Invitrogen, Burlington ON, Canada) vector and were sequenced by CRCHUQ/CHUL sequencing platform (Québec Qc, Canada). The DR3 sequences were compared with those found in the BLAST database and analyzed with the Human Genome Browser Gateway http://genome.ucsc.edu/cgi-bin/hgGateway and the AceView genes http://www.ncbi.nlm.nih.gov/IEB/Research/Acembly/ databases.

### Analysis of DR3 variants

Total RNA was extracted from (1 × 10^6^) cells using Qiagen RNeasy kit and one μg was used for a reverse transcription using Omniscript reverse transcriptase (Qiagen). Then, the full length DR3 was amplified by PCR using Qiagen Hotstart polymerase and the following primers: 5'-CGTCGGAGGGCTATGGAGCAGC and 5'-GGCCGGCTGGTGCTGCTACGC following the manufacturer's instructions. Thereafter, the region from exon 5 to exon 7 of DR3 was amplified by PCR, as previously described, using DR3 full length PCR product as a template and the following primers: 5'-CCCGCAGAGATACTGACTGTGGGAC and 5'-GTAGCCAGGGGTCCAGCTGTTACC. The resulting products were separated by agarose gel electrophoresis.

For more precise quantification, targeted PCR reactions were carried out, and the amplified products were analyzed by automated chip-based microcapillary electrophoresis on an Agilent 2100 Bioanalyzer instrument (Agilent Technologies, Santa Clara, CA) as previously described [37]. Amplicon sizing and relative quantification was performed by the manufacturer's software. The primers used were Forward: 5'-TTCCCGCAGAGA TACTGACTG and Reverse: 5'-AGCACCTGG ACCCAGAACA.

### Western blotting

Cells lysis was done at 4°C in extraction buffer (60 mM Tris-base, pH 6.8, 10% glycerol, and 3% sodium dodecyl sulphate) added with 5% β-mercaptoethanol just before use. Then, lysates were boiled, vortexed twice and centrifuged at 13,000 *g *for 5 minutes. Proteins were separated by SDS-PAGE and transferred to a nitrocellulose membrane. Each antibody was used according to the manufacturer's protocol. Blots were then revealed with Super signal West pico kit obtained from Pierce Biotechnology Inc (Rockford IL). If necessary, the membrane was reprobed for normalization.

### Apoptosis evaluation

**1) ***by DNA fragmentation*. HT29 cells were treated with rhE-Selectin/Fc at 10 μg/ml for 4 hours or 24 hours, or were treated with phenethyl isothiocyanate at 50 μM for 24 hours. Cells were washed twice with PBS, fixed with 3,7% formaldehyde and stained with Hoechst for 60 min at room temperature in the dark. The cells were examined with a Nikon Eclipse 800 equipped with a 40 × objective lens. **2) ***by caspase activation*. Caspase 8 and 3 activities were evaluated by western blotting using anti-caspase 8 and anti active-caspase-3 antibodies. The assays were performed on pools of cells containing both floating and adhering cells.

## Results and Discussion

### Death receptor-3 mediates the adhesion of colon cancer cells to endothelial cells expressing E-selectin under flow conditions

We previously reported that the adhesion of HT29 colon cancer cells to endothelial cells under static conditions is mediated by the binding interaction between DR3 expressed by cancer cells and E-selectin expressed by endothelial cells [23,38]. Considering that the adhesion of cancer cells to the endothelium *in vivo *occurs under flow and shear stress conditions, we ascertained the role of DR3 in mediating adhesion of colon cancer cells to E-selectin under flow conditions using a laminar flow chamber.

**Figure 1:**
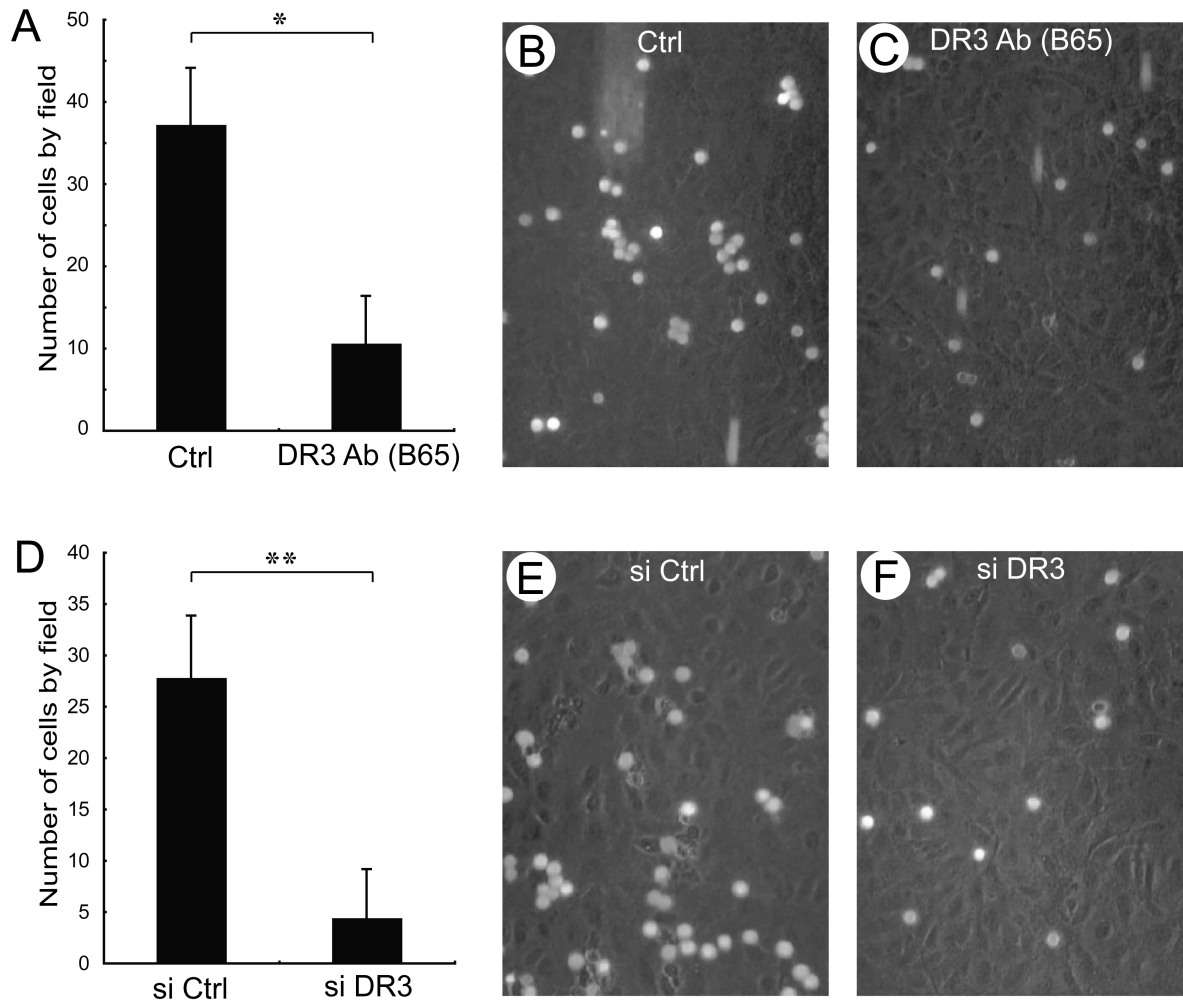
**DR3 mediates the adhesion of colon cancer cells to E-selectin-expressing endothelial cells in a laminar flow chamber**. **A to C**. HUVECs were stimulated with 20 ng/mL of IL-1β for 4 h. Live HT29 cells (2 × 10^6 ^per assay) stained with Calcein AM were treated with anti-DR3 blocking antibody (B65) or with a control irrelevant antibody (Ctrl: MOPC21) for 30 min prior to being introduced in the circulating medium. **D to F**. The knockdown of DR3 expression in HT29 was performed by small interfering RNA (siDR3). siRNA targeting unrelated mRNAs was used as control (Ctrl). Following treatment, HT29 cells were placed in a laminar flow chamber under a shear stress of 1 dyne/cm^2 ^as in **A**. In **A **and **D**, the cells attached to the endothelium were counted after 25 min in more than 5 fields per condition. *p < 0.01 (Student t test using the IL-1β condition as reference). In **B, C, E **and **F**, representative fields are shown.

HUVEC forming a tight monolayer on gelatin-coated glass slides were treated or not for 4 hours with IL-1β to induce the expression of E-selectin. Then, the cultures were placed in a laminar flow chamber in which medium circulated under a flow that gave a physiological shear stress of 1 dyne/cm^2 ^[9]. Live HT29 cells (2 × 10^6 ^per assay) stained with Calcein AM and pre-treated or not with anti-DR3 antibody or an siRNA that knocks down the expression of DR3 were injected in the flow system and video sequences were taken at 25 minute intervals. The cells attached to the endothelium were counted in more than 5 fields per condition. Results showed that, after the first 25 min, no HT29 cancer cell adhered to endothelial cells that did not express E-selectin. However, they adhered in a time-dependent manner to HUVEC expressing E-selectin and the adhesion was blocked by treating the endothelial layer with an anti-Eselectin antibody (data not shown and [9]). These findings clearly indicated that the adhesion of HT29 cells to endothelial cells was E-selectin-dependent. As shown in Figure 1A-F**(and **additional files 1, 2, 3 and 4), the adhesion was also DR3-dependent given that inhibiting DR3 with the anti-DR3 antibody or knocking down its expression with siRNA led to a 7-fold reduction of the adhesion of HT29 cells to HUVEC expressing E-selectin.

These results suggest that the adhesion of colon cancer cells in blood circulation relies mainly on DR3/E-selectin interaction. In a previous study, we described three distinct mechanisms by which circulating cancer cells interact with E-selectin to initiate transendothelial migration: formation of a mosaic between cancer cells and endothelial cells, paracellular diapedesis at the junction of three endothelial cells, and transcellular diapedesis [9]. The results of the present study now suggest that DR3 expressed by colon cancer cells is a major partner of E-selectin in inducing these mechanisms of diapedesis *in vivo*. In particular, it is possible that DR3 binding to E-selectin is the initial event that activates E-selectin oligomerization and thereby ERK-mediated disruption of the adherent junctions and diapedesis [38]. Another possibility is that the DR3/E-selectin binding triggers the release of chemokines or cytokines, such as VEGF, by endothelial cells or cancer cells, which later triggers diapedesis [39,40].

### E-selectin does not induce apoptosis in HT29 cells

**Figure 2:**
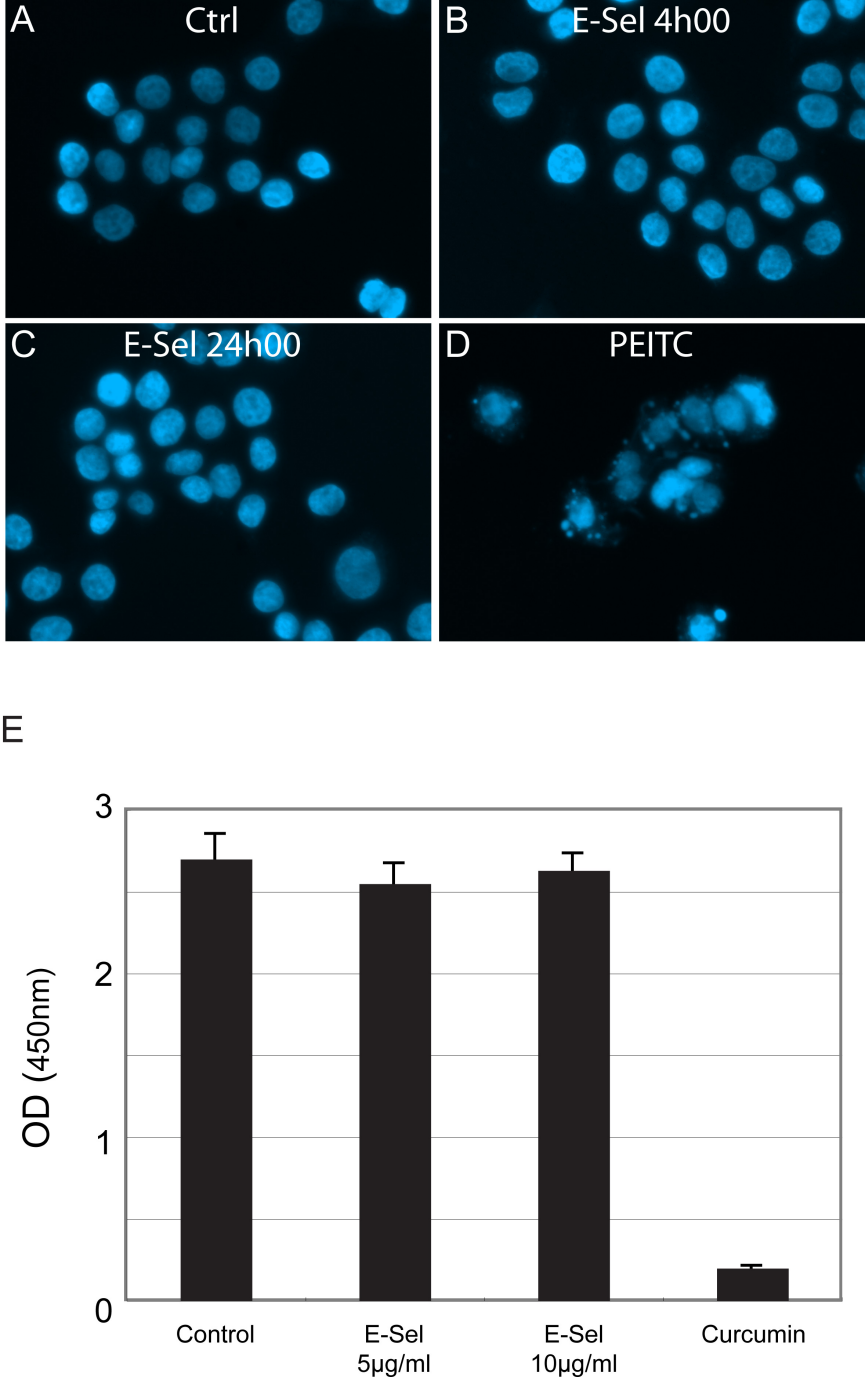
**E-selectin does not induce apoptosis in HT29 cells**. **A **to **D**, HT29 cells were left untreated (**A**) or were treated with E-selectin at 10 μg/ml for 4 hours (**B**) or 24 hours (**C**), or were treated with phenethyl isothiocyanate at 50 μM for 24 hours (**D**). Cells were fixed and stained with Hoechst dye and examined for nuclear fragmentation. In **E**, 24 hours after being plated, HT29 cells were left to grow for 96 hours with or without E-selectin or the apoptosis inducer curcumin (75 μM). At the end of the treatments, the cell survival was evaluated by their ability to convert tetrazolium salt into formozan (WST-1 assay), which was monitored at 450 nm.

DR3 is a member of the TNF receptor family whose activation is typically associated with apoptosis [24]. Along these lines, the ectopic expression of DR3 in HEK293 or HeLa cells induced marked apoptosis [30]. Accordingly, we next investigated whether the activation of DR3 by E-selectin triggers apoptosis. We found that chimeric rhE-selectin/Fc taken as ligand did not induce apoptosis in HT29 cells, even at concentrations twice as those required to induce DR3-mediated activation of p38 [23]. This is illustrated in Figure 2A-C which shows that rhE-selectin/Fc at a concentration of 10 μg/ml did not induce nuclear fragmentation even after 24 h exposure. In contrast, phenylethyl isothiocyanate, a death receptor-independent inducer of apoptosis in these cells exerted a strong apoptotic response [41,42] (Figure 2D). Consistent with these findings, we found that E-selectin, in contrast to curcumin, did not reduce cell survival even after 96 h of exposure, as determined by the WST-1 assay (Figure 2E). In the *in vivo *context, these results suggest that the DR3-mediated adhesion of colon cancer cells to endothelial cell E-selectin may trigger activation of survival pathways in cancer cells that impair apoptosis.

### E-selectin-induced activation of Death receptor-3 triggers the activation of PI3K in a Src kinase-dependent manner

**Figure 3:**
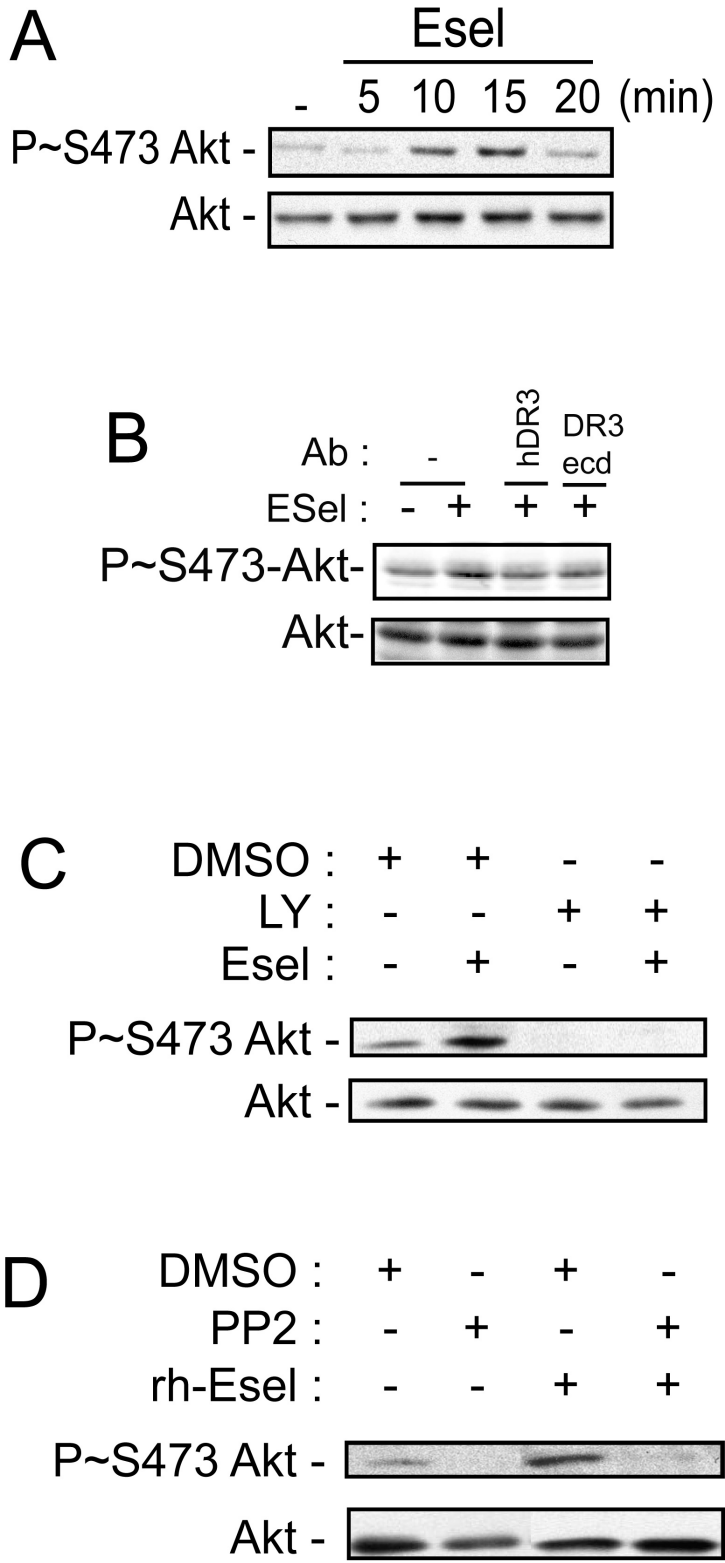
**E-selectin mediates Src kinase-dependent phosphorylation of Akt through DR3**. **A**) HT29 cells were left untreated or treated for different periods of time (5, 10,15 and 20 min) with rhE-selectin/Fc (5 μg/ml). The proteins were extracted and separated by SDS-PAGE and transferred onto a nitrocellulose membrane. Akt activation was analysed by western blotting with specific anti-phospho (Ser 473)-Akt antibody. The blots was then stripped and reprobed with antibody recognizing total Akt as loading control. **B**) HT29 cells were pre-incubated or not for 30 minutes with anti-DR3 (hDR3) or anti-DR3 (DR3ecd) before being incubated with rhE-selectin/Fc (5 μg/ml for 15 min). The proteins were extracted and processed as in **A**. **C**) HT29 cells were pre-treated for 2 hours with DMSO (0.1%) or LY249002 (20 μM) before being incubated with rhE-selectin/Fc (5 μg/ml for 15 min). The proteins were extracted and processed as in **A**. **D**) HT29 cells were pretreated for 2 hours with DMSO (0.08%) or PP2 (10 μM) to inhibit Src family kinases. Thereafter, the cells were left untreated or treated with E-selectin (5 μ/ml for 15 min). The proteins were extracted and processed as in **A**.

Inhibition of ERK is associated with a weak increase in the activation of caspase-3 in LoVo colon cancer cells treated by rhE-selectin/Fc [23]. This suggests that another pathway is involved in conferring resistance to apoptosis to colon cancer cells adhering to E-selectin. We thus evaluated the contribution of the PI3K pathway given that it is a major pro-survival pathway. By measuring the phosphorylation of AKT at Ser473 (Figure 3A), we found that exposure of HT29 cells to rhE-selectin/Fc induced a time-dependent activation of PI3K which peaked at 15 min. The activation of PI3K by E-selectin is dependent on DR3 activation given that it was abolished by two DR3 neutralizing antibodies (Figure 3B). Interestingly, the E-selectin-induced phosphorylation of Akt at Ser473 was sensitive to LY294002, a well-known inhibitor of PI3K activity (Figure 3C). In line with the findings that showed that PI3K activation was downstream of Src in response to different cytokines including TNFα, we found that the phosphorylation of Akt at Ser473 was also sensitive to Src inhibition by the pan Src inhibitor PP2 (Figure 3D)[43,44]. Interestingly, DR3 contains an ITAM motif within its death domain that harbors two tyrosine residues (Y376 and Y394) that have been suggested to be phosphorylated via Src activation [45]. In light of our results, it is thus possible that Src-dependent activation of the PI3K pathway may originate from an Src-mediated phosphorylation of one of these tyrosines. Hence, these findings suggest that E-selectin-mediated activation of Src may trigger phosphorylation of DR3 which would converge on the activation of the PI3K pathway, a major regulator of cell survival [46]. Accordingly, we next investigated the signaling events by which the activation of PI3K downstream of DR3 may mediate the survival of colon cancer cells.

### The activation of PI3K downstream of DR3 induces the activation of NFκB

**Figure 4:**
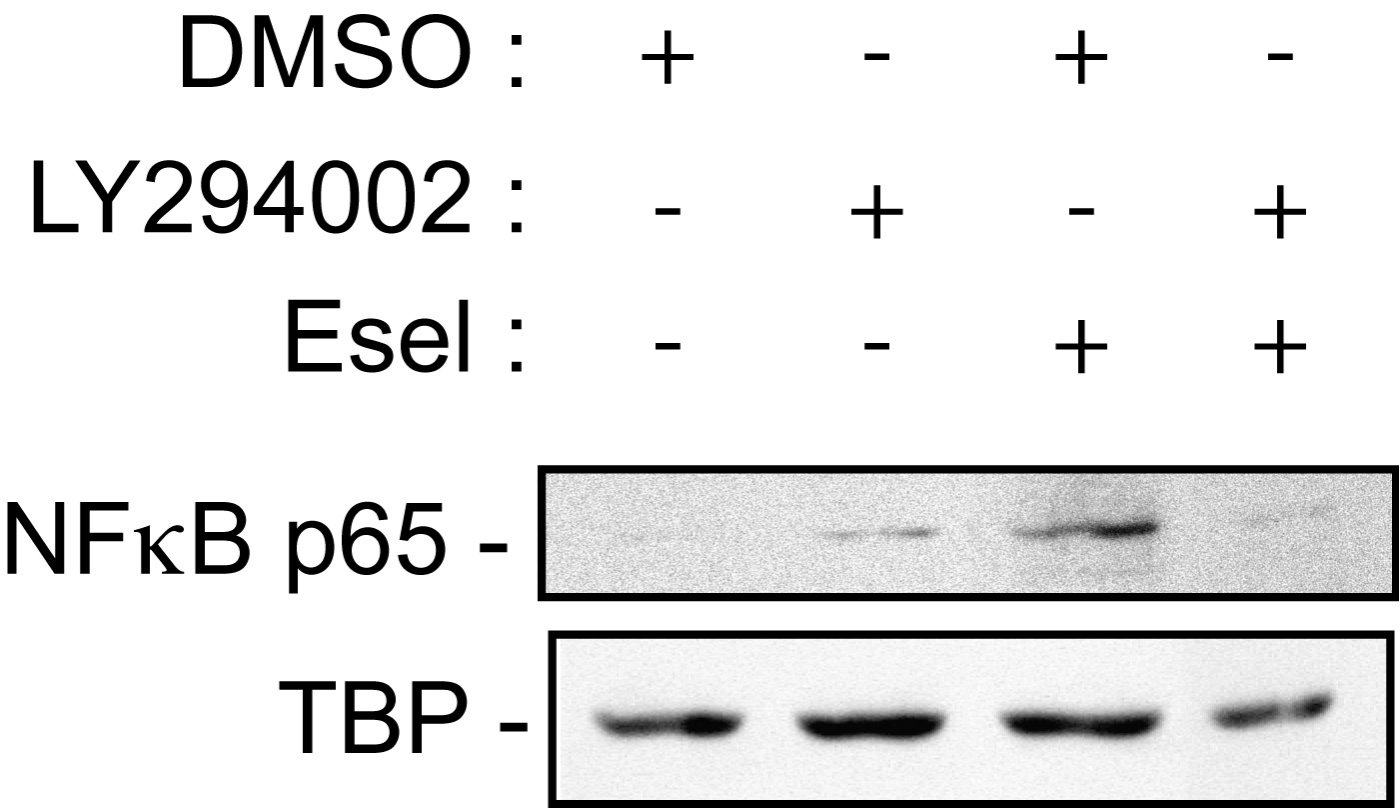
**DR3 activation triggers NFκB activation through PI3K**. HT29 cells were pre-treated for 2 hours with PI3K inhibitor LY294002 (20 μM) or DMSO as vehicle (0.1%) and treated or not with rhE-selectin/Fc (5 ng/ml for 15 min). Nuclear proteins were extracted and processed for western blotting using specific antibodies for total p65 NFκB or for TATA binding protein (TBP) used as nuclear protein control.

**Figure 5:**
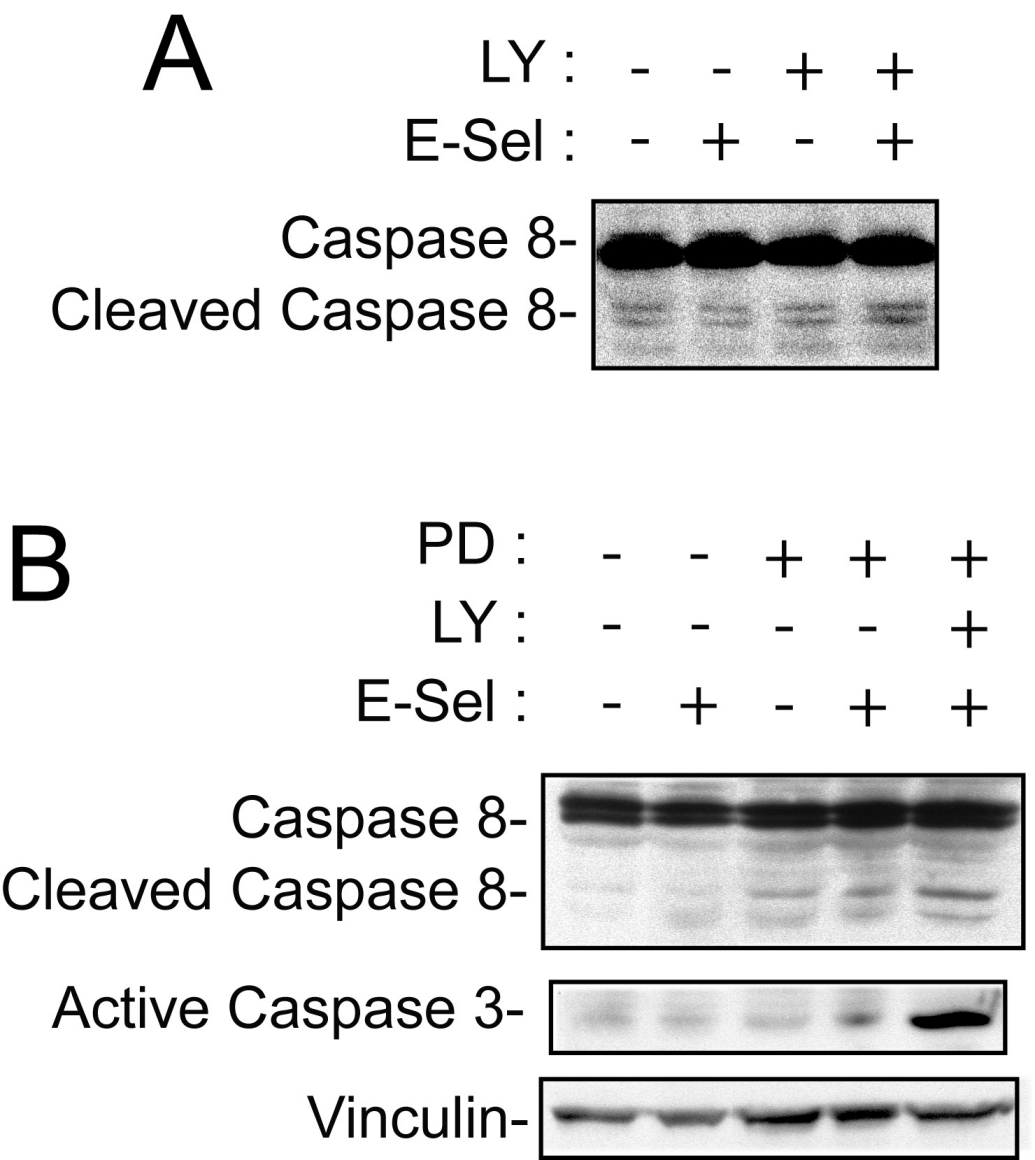
**PI3K and ERK activation impairs caspase activation**. **A**) HT29 cells were pre-treated for 30 minutes with DMSO (0.1%) or LY 249002 (20 μM) before being incubated with rhE-selectin/Fc (5 μg/ml for 24 hours). The proteins were extracted and were separated by SDS-PAGE and transferred onto a nitrocellulose membrane. Caspase-8 cleavage/activation was analysed by western blotting using specific anti-caspase-8 antibody. **B**) HT29 cells were pre-treated with MEK inhibitor PD059098 (50 μM for 60 min) with or without PI3K inhibitor LY294002 (20 μM for 30 min) or DMSO as vehicle (0.1% for 60 min) and treated or not with rhE-selectin/Fc (5 ng/ml for 24 hours). The proteins were extracted and were separated by SDS-PAGE and transferred onto a nitrocellulose membrane. Caspase-3 activation was analysed by western blotting using a specific anti-active-caspase-3 antibody and a specific antibody against vinculin as loading control.

Earlier findings have highlighted the point that, depending on cell types and cellular context, DR3 activation was associated either with apoptosis following the recruitment of the apoptotic cascade on the death domain, or survival following activation of the pro-survival factor NFκB [31]. Hence, we next investigated the status of NFκB following activation of DR3 by E-selectin. As shown in Figure 4, we found that E-selectin induced a LY294002-sensitive and thereby PI3K-dependent activation of NFκB, as evaluated by the translocation of NFκB-p65 subunit into the nucleus (Figure 4). Previous studies have reported that NFκB was activated by DR3 and other TNFR following the activation of NFκB-inducing kinase downstream of the recruitment of TRAF2 to the receptor death domain [47,48]. In turn, this leads to increased survival [29,31,49]. Here our findings suggest that the activation of NFκB downstream of DR3 may be independent of the TRAF2 pathway and would depend on the activation of the PI3K/Akt pathway, presumably downstream of a Src-dependent tyrosine phosphorylation of DR3 within the ITAM motif [50,51]. This possibility is in line with the finding that cell survival downstream of CD95/Fas is associated with its tyrosine phosphorylation, upstream of the activation of the PI3K/AKT pathway [26]. Consistent with a role of PI3K/NFκB pathways in protecting HT29 cells from apoptosis in response to E-selectin, we further found that the inhibition of PI3K by LY294002 increased the cleavage of caspase 8 in response to E-selectin (Figure 5A). We previously reported that ERK contributes to protect colon cancer cells from apoptosis following activation of DR3 by E-selectin [23]. Accordingly, the co-inhibition of both ERK and PI3K, respectively by PD098059 and LY294002, was associated with a synergistic activation of both caspase-8 and caspase-3 in response to E-selectin (Figure 5B). This result indicates that both ERK and the PI3K/Akt//NFκB axis contribute to confer apoptosis resistance to colon cancer cells in response to E-selectin. In addition, it confirms the pro-survival function of the ERK pathway downstream of DR3, as we previously reported [23].

### Metastatic colon cancer cells express transmembrane and cytoplasmic deletants of DR3

**Figure 6:**
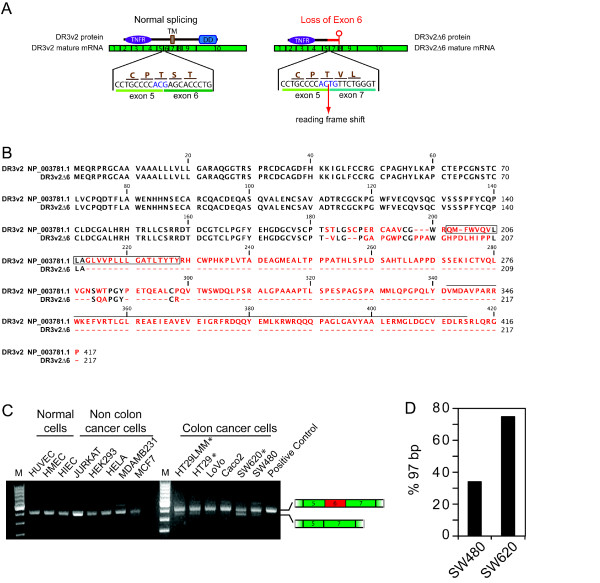
**Colon carcinoma cells HT29 express a splice variant of DR3 missing the exon 6**. **A) **Schematic representation of the normal splicing and the splicing associated with the loss of exon 6 within DR3v2. **B) **Predicted amino acids sequence of DR3v2 deleted of exon 6. The transmembrane domain is shown in the black box and the black line represents the death domain. Identical amino acid sequences are in black and non-identical are in red. GeneBank sequence NP_003781.1 was used as reference for DR3v2. **C) **Total RNA from different cell lines were extracted and were amplified by reverse transcriptase. Primers were designed to amplify by PCR the region from exon 5 to exon 7 of DR3v2. The resulting products were run through agarose gel. **D) **Total RNA from SW480 or SW620 cell lines were extracted and were amplified by reverse transcriptase. PCR reactions were carried out using specific primers amplifying region around exon 6 which produce a 97bp band for the DR3v2Δ6. Automated sizing and relative quantification of the amplicon versus total amplicons was performed using the manufacturer's software.

Next, we verified whether a mutation in DR3 could further contribute to the lack of apoptosis induced by E-selectin. By using PCR approach, we cloned and sequenced DR3 cDNA, and we found major variations in the expression profile of DR3. From 36 different clones, we discovered that, in addition to the full-length version of DR3, HT29 cells expressed splice variants of DR3. One of them is characterized by a loss of exon 6 (DR3Δ6) (Additional file 5). The joint between the last two nucleotides of exon 5 and the first two nucleotides of exon 7 leads to a shift in the reading frame introducing a premature stop codon, located at the beginning of exon 8 (Figure 6A). This variant codes for a new protein whose last 37 amino acids are not found in any of the known variants of DR3. This protein has no trans-membrane and death domain (Figure 6B) and thus is unable to trigger apoptosis. Interestingly, by PCR amplification of the region around exon 6, we found that the relative proportion of DR3Δ6 was higher in metastatic colon cancer cells (HT29, HT29LMM, SW620) in comparison to normal colon epithelial cells (HIEC) and endothelial cells (HUVEC, HMEC), as well as in metastatic cancer cells that are not of colon origin (Jurkat, Hela, MCF7, MDAMB231) (Figure 6C). Notably, it is particularly clear that the relative level of of DR3Δ6 to full length DR3 is higher in metastatic SW620 cells relative to non-metastatic SW480 cells taken from the primary tumor site of the same patient. In fact, more precise quantification by targeted PCR reactions and analysed of the amplified products by chip-based microcapillary electrophoresis indicated that the ratio of DR3Δ6 to full length DR3 doubled in SW620 cells relative to SW480 (Figure 6D). These findings strongly suggest that the expression of DR3Δ6 is associated with a metastatic phenotype in colon cancer. In turn, this raises the possibility that, during the acquisition and progression of malignancy, colon cancer cells evolved to develop alternative splicing mechanisms favoring the shifting of a death-receptor toward a survival receptor. Along these lines, it was shown that a variant of DR3, (DR3β), differs from the described DR3 isoform 2 by the inclusion of a 28 amino-acid stretch in the extracellular domain. Whereas DR3 was expressed in all the cell lines and lymphoma samples tested, DR3β expression was restricted to lymphoid T-cell and immature B-cell lines and to some cases of follicular lymphoma. This is consistent with our finding that different isoforms of DR3 can contribute to cancer [28]. It is difficult at present to fully understand the mechanism of alternative splicing regulation acting on DR3. One possibility relies on the phosphorylation of serine-arginine rich proteins (SRPs) known to be major regulators of alternative splicing in colon cancer cells [52]. This is further supported by the fact that PI3K which is activated by E-selectin-mediated stimulation of DR3 also regulates the phosphorylation of SRPs [53]. Interestingly, death decoy receptor-3 (DcR3), another member of the TNF receptor superfamily, is a soluble receptor that is highly expressed in various tumors including colon cancer and that act as a negative regulator of DR3[54]. Although, DR3Δ6 differs in sequence from DcR3, it is possible that it also acts as a decoy receptor for the activation of DR3 by E-selectin.

## Conclusion

Overall, our study reveals that activation of DR3 by E-selectin in HT29 cells leads to the activation of the PI3K/NFκB survival pathway. This results in cells that are both resistant to apoptosis and which have acquired an increased capacity to survive. We also found that HT29 cells have developed alternative splicing mechanisms that favor the shift of DR3 from a full length signaling receptor to deletants devoid of death domain and thus unable to trigger apoptosis. This is the first time that such a bi-functional insidious mechanism is reported to confer metastatic properties to colon cancer cells.

## List of abbreviations used

Akt: v-akt murine thymoma viral oncogene homologue 1; DR3: death receptor 3; ERK: extracellular-signal regulated kinase; FADD: Fas-associated protein with death domain; MAPK: mitogen-activated protein kinase; NFkB: nuclear factor kappa light chain in activated B cells; RIP: receptor-interacting protein; PI3K: phosphatidylinositol-3 kinase; TL1A: TNF ligand-related molecule 1a; TNF: tumor necrosis factor; TRADD: tumor necrosis factor receptor type 1-associated DEATH domain protein; VEGI: vascular endothelial growth inhibitor.

## Author information

Nicolas Porquet, Andrée Poirier, François Houle, Anne-Laure Pin, Stéphanie Gout, Pierre-Luc Tremblay, Éric Paquet and Jacques Huot are from « Le Centre de recherche en cancérologie de l'Université Laval et Centre de recherche du CHUQ, l'Hôtel-Dieu de Québec, 9 rue McMahon, Québec G1R 2J6 Canada ». Nicolas Porquet is now at « The Institute of Developmental Biology and Cancer, CNRS UMR6543, Université Nice Sophia Antipolis 06108 Nice Cedex 2, France ». Stéphanie Gout is now at « Le Centre de recherche Inserm/UJF U823 Equipe 2, Institut Albert Bonniot BP 170, 38052 Grenoble Cedex 09, France ». François A Auger and Pierre-Luc Tremblay are both from « Le Laboratoire d'Organogenèse Expérimentale, Centre hospitalier affilié Universitaire de Québec, 1401, 18^e ^rue Québec, G1J 1Z4, Canada ». Roscoe Klinck is from « Le Laboratoire de Génomique Fonctionnelle de l'Université de Sherbrooke, 3201, rue Jean Mignault, Sherbrooke J1E 4K8 Canada ».

## Competing interests

The authors declare that they have no competing interests.

## Authors' contributions

NP, AP and FH have performed the signaling and survival experiments. ALP and AP have performed PCR assays and NP, AP and ALP have equally contributed to the identification of DR3Δ6. RK did the quantification of the DR3 variants. SG, PLT and FAA realized the experiments done in flow chambers. EP contributed to the statistics and bioinformatics analysis. FH finalized the figures. NP and JH wrote the manuscript with the contribution of the other authors. All authors read and approved the final version.

## Pre-publication history

The pre-publication history for this paper can be accessed here:

http://www.biomedcentral.com/1471-2407/11/285/prepub

## Supplementary Material

Additional file 1 **An irrelevant antibody to DR3 does not influence the adhesion of colon cancer cells to E-selectin-expressing endothelial cells in a laminar flow chamber**. HUVECs were trypsinized and grown for 24 hrs on gelatin-coated slides. These endothelial cells were treated with 20 ng/ml IL-1β for 4 h to induce the expression of E-selectin. The endothelial cell cultures were then placed in a laminar flow chamber under a shear stress of 1 dyne/cm^2^. Live HT29 cells (2 × 10^6 ^per assay) stained with Calcein AM were treated with a control irrelevant antibody (Ctrl: MOPC21) for 30 min prior to being introduced in the medium circulating over endothelial cells. Videos were taken directly using a camera mounted on a TE2000 fluorescence microscope at ×20 magnification to follow the adhesion of HT-29 cells to HUVECs. Click here for file

Additional file 2 **An anti-DR3 blocking antibody impairs the adhesion of colon cancer cells to E-selectin-expressing endothelial cells in a laminar flow chamber**. HUVECs were trypsinized and grown for 24 hrs on gelatin-coated slides. These endothelial cells were treated with 20 ng/ml IL-1β for 4 h to induce the expression of E-selectin. The endothelial cell cultures were then placed in a laminar flow chamber under a shear stress of 1 dyne/cm^2^. Live HT29 cells (2 × 10^6 ^per assay) stained with Calcein AM were treated with an anti-DR3 blocking antibody (B65) for 30 min prior to being introduced in the medium circulating over endothelial cells. Videos were taken directly using a camera mounted on a TE2000 fluorescence microscope at ×20 magnification to follow the adhesion of HT-29 cells to HUVECs. Click here for file

Additional file 3 **An irrelevant control siRNA does not influence the adhesion of colon cancer cells to E-selectin-expressing endothelial cells in a laminar flow chamber**. HUVECs were trypsinized and grown for 24 hrs on gelatin-coated slides. These endothelial cells were treated with 20 ng/ml IL-1β for 4 h to induce the expression of E-selectin. The endothelial cell cultures were then placed in a laminar flow chamber under a shear stress of 1 dyne/cm^2^. Live HT29 cells were transfected by electroporation with control siRNA purchased from Qiagen. Tumor cells in suspension (2 × 10^6 ^per assay) were labeled for 30 min with Calcein AM and washed twice with M199 medium before being added in the medium circulating over endothelial cells. Videos were taken directly using a camera mounted on a TE2000 fluorescence microscope at ×20 magnification to follow the adhesion of HT-29 cells to HUVECs. Click here for file

Additional file 4 **Knocking down the expression of DR3 with a human DR3 siRNA impairs the adhesion of colon cancer cells to E-selectin-expressing endothelial cells in a laminar flow chamber**. HUVECs were trypsinized and grown for 24 hrs on gelatin-coated slides. These endothelial cells were treated with 20 ng/ml IL-1β for 4 h to induce the expression of E-selectin. The endothelial cell cultures were then placed in a laminar flow chamber under a shear stress of 1 dyne/cm^2^. Live HT29 cells were transfected by electroporation with human DR3 siRNA purchased from Qiagen. Tumor cells in suspension (2 × 10^6 ^per assay) were labeled for 30 min with Calcein AM and washed twice with M199 medium before being added in the medium circulating over endothelial cells. Videos were taken directly using a camera mounted on a TE2000 fluorescence microscope at ×20 magnification to follow the adhesion of HT-29 cells to HUVECs. Click here for file

Additional file 5 **Sequence alignment between Dr3v2 and splice variant of DR3 missing the exon 6**. Total RNA from HT29 cells were extracted and were amplified by reverse transcriptase. The resulting cDNAs were amplified by PCR using primers that bind to all DR3 isoforms outside of the reading frame. A second round of PCR was done using a 3' primer outside the reading frame and a 5' primer just inside the reading frame. These final cDNAs were cloned and then sequenced. The sequence for the DR3v2 deleted of exon 6 is shown below the reference sequence NM_003790.2. Grey colored box sequences and blank sequences represent alternating exons. Click here for file
