# Supplementary material for: Survival advantages conferred to colon cancer cells by E-selectin-induced activation of the PI3K-NFκB survival axis downstream of Death receptor-3
Source: BMC Cancer. 2011 Jul 1;11:285. doi: 10.1186/1471-2407-11-285 (PMC3177907; doi:10.1186/1471-2407-11-285)
Supplement: Additional file 5 — Sequence alignment between Dr3v2 and splice variant of DR3 missing the exon 6. Total RNA from HT29 cells were extracted and were amplified by reverse transcriptase. The resulting cDNAs were amplified by PCR using primers that bind to all DR3 isoforms outside of the reading frame. A second round of PCR was done using a 3' primer outside the reading frame and a 5' primer just inside the reading frame. These final cDNAs were cloned and then sequenced. The sequence for the DR3v2 deleted of exon 6 is shown below the reference sequence NM_003790.2. Grey colored box sequences and blank sequences represent alternating exons. [file 1471-2407-11-285-S5.pdf]

```
                                          20                                       40                                       60
DR3v2 NM_003790.2  CGGGCCCTGC GGGCGCGGGG CTGAAGGCGG AACCACGACG GGCAGAGAGC ACGGAGCCGG GAAGCCCCTG   70
          DR3v2Δ6  ---------- ---------- ---------- ---------- ------GAGC ACGGAGCCGG GAAGCCCCTG   24

                               80                       100                       120                       140
DR3v2 NM_003790.2  GGCGCCCGTC GGAGGGCTAT GGAGCAGCGG CCGCGGGGCT GCGCGGCGGT GGCGGCGGCG CTCCTCCTGG  140
          DR3v2Δ6  GGCGCCCGTC GGAGGGCTAT GGAGCAGCGG CCGCGGGGCT GCGCGGCGGT GGCGGCGGCG CTCCTCCTGG   94

                                          160                                      180                                      200
DR3v2 NM_003790.2  TGCTGCTGGG GGCCCGGGCC CAGGGCGGCA CTCGTAGCCC CAGGTGTGAC TGTGCCGGTG ACTTCCACAA  210
          DR3v2Δ6  TGCTGCTGGG GGCCCGGGCC CAGGGCGGCA CTCGTAGCCC CAGGTGTGAC TGTGCCGGTG ACTTCCACAA  164

                               220                      240                       260                       280
DR3v2 NM_003790.2  GAAGATTGGT CTGTTTTGTT GCAGAGGCTG CCCAGCGGGG CACTACCTGA AGGCCCCTTG CACGGAGCCC  280
          DR3v2Δ6  GAAGATTGGT CTGTTTTGTT GCAGAGGCTG CCCAGCGGGG CACTACCTGA AGGCCCCTTG CACGGAGCCC  234

                                          300                                      320                                      340
DR3v2 NM_003790.2  TGCGGCAACT CCACCTGCCT TGTGTGTCCC CAAGACACCT TCTTGGCCTG GGAGAACCAC CATAATTCTG  350
          DR3v2Δ6  TGCGGCAACT CCACCTGCCT TGTGTGTCCC CAAGACACCT TCTTGGCCTG GGAGAACCAC CATAATTCTG  304

                               360                      380                       400                       420
DR3v2 NM_003790.2  AATGTGCCCG CTGCCAGGCC TGTGATGAGC AGGCCTCCCA GGTGGCGCTG GAGAACTGTT CAGCAGTGGC  420
          DR3v2Δ6  AATGTGCCCG CTGCCAGGCC TGTGATGAGC AGGCCTCCCA GGTGGCGCTG GAGAACTGTT CAGCAGTGGC  374

                                          440                                      460                                      480
DR3v2 NM_003790.2  CGACACCCGC TGTGGCTGTA AGCCAGGCTG GTTTGTGGAG TGCCAGGTCA GCCAATGTGT CAGCAGTTCA  490
          DR3v2Δ6  CGACACCCGC TGTGGCTGTA AGCCAGGCTG GTTTGTGGAG TGCCAGGTCA GCCAATGTGT CAGCAGTTCA  444

                               500                      520                       540                       560
DR3v2 NM_003790.2  CCCTTCTACT GCCAACCATG CCTAGACTGC GGGGCCCTGC ACCGCCACAC ACGGCTACTC TGTTCCCGCA  560
          DR3v2Δ6  CCCTTCTACT GCCAACCATG CCTAGACTGC GGGGCCCTGC ACCGCCACAC ACGGCTACTC TGTTCCCGCA  514

                                          580                                      600                                      620
DR3v2 NM_003790.2  GAGATACTGA CTGTGGGACC TGCCTGCCTG GCTTCTATGA ACATGGCGAT GGCTGCGTGT CCTGCCCCAC  630
          DR3v2Δ6  GAGATACTGA CTGTGGGACC TGCCTGCCTG GCTTCTATGA ACATGGCGAT GGCTGCGTGT CCTGCCCCAC  584

                               640                      660                       680                       700
DR3v2 NM_003790.2  GAGCACCCTG GGGAGCTGTC CAGAGCGCTG TGCCGCTGTC TGTGGCTGGA GGCAGATGTT CTGGGTCCAG  700
          DR3v2Δ6  ---------- ---------- ---------- ---------- ---------- ------TGTT CTGGGTCCAG  598

                                          720                                      740                                      760
DR3v2 NM_003790.2  GTGCTCCTGG CTGGCCTTGT GGTCCCCCTC CTGCTTGGGG CCACCCTGAC CTACACATAC CGCCACTGCT  770
          DR3v2Δ6  GTGCTCCTGG CTGGCCTTGT GGTCCCCCTC CTGCTTGGGG CCACCCTGAC CTACACATAC CGCCACTGCT  668

                               780                      800                       820                       840
DR3v2 NM_003790.2  GGCCTCACAA GCCCCTGGTT ACTGCAGATG AAGCTGGGAT GGAGGCTCTG ACCCCACCAC CGGCCACCCA  840
          DR3v2Δ6  GGCCTCACAA GCCCCTGGTT ACTGCAGATG AAGCTGGGAT GGAGGCTCTG ACCCCACCAC CGGCCACCCA  738

                                          860                                      880                                      900
DR3v2 NM_003790.2  TCTGTCACCC TTGGACAGCG CCCACACCCT TCTAGCACCT CCTGACAGCA GTGAGAAGAT CTGCACCGTC  910
          DR3v2Δ6  TCTGTCACCC TTGGACAGCG CCCACACCCT TCTAGCACCC CCTGACAGCA GTGAGAAGAT CTGCACCGTC  808

                               920                      940                       960                       980
DR3v2 NM_003790.2  CAGTTGGTGG GTAACAGCTG GACCCCTGGC TACCCCGAGA CCCAGGAGGC GCTCTGCCCG CAGGTGACAT  980
          DR3v2Δ6  CAGTTGGTGG GTAACAGCTG GACCCCTGGC TACCCCGAGA CCCAGGAGGC GCTCTGCCCG CAGGTGACAT  878

                                          1,000                                    1,020                                    1,040
DR3v2 NM_003790.2  GGTCCTGGGA CCAGTTGCCC AGCAGAGCTC TTGGCCCCGC TGCTGCGCCC ACACTCTCGC CAGAGTCCCC 1050
          DR3v2Δ6  GGTCCTGGGA CCAGTTGCCC AGCAGAGCTC TTGGCCCCGC TGCTGCGCCC ACACTCTCGC CAGAGTCCCC  948

                               1,060                    1,080                     1,100                     1,120
DR3v2 NM_003790.2  AGCCGGCTCG CCAGCCATGA TGCTGCAGCC GGGCCCGCAG CTCTACGACG TGATGGACGC GGTCCCAGCG 1120
          DR3v2Δ6  AGCCGGCTCG CCAGCCATGA TGCTGCAGCC GGGCCCGCAG CTCTACGACG TGATGGACGC GGTCCCAGCG 1018

                                          1,140                                    1,160                                    1,180
DR3v2 NM_003790.2  CGGCGCTGGA AGGAGTTCGT GCGCACGCTG GGGCTGCGCG AGGCAGAGAT CGAAGCCGTG GAGGTGGAGA 1190
          DR3v2Δ6  CGGCGCTGGA AGGAGTTCGT GCGCACGCTG GGGCTGCGCG AGGCAGAGAT CGAAGCCGTG GAGGTGGAGA 1088

                               1,200                    1,220                     1,240                     1,260
DR3v2 NM_003790.2  TCGGCCGCTT CCGAGACCAG CAGTACGAGA TGCTCAAGCG CTGGCGCCAG CAGCAGCCCG CGGGCCTCGG 1260
          DR3v2Δ6  TCGGCCGCTT CCGAGACCAG CAGTACGAGA TGCTCAAGCG CTGGCGCCAG CAGCAGCCCG CGGGCCTCGG 1158

                                          1,280                                    1,300                                    1,320
DR3v2 NM_003790.2  AGCCGTTTAC GCGGCCCTGG AGCGCATGGG GCTGGACGGC TGCGTGGAAG ACTTGCGCAG CCGCCTGCAG 1330
          DR3v2Δ6  AGCCGTTTAC GCGGCCCTGG AGCGCATGGG GCTGGACGGC TGCGTGGAAG ACTTGCGCAG CCGCCTGCAG 1228

                               1,340                    1,360                     1,380                     1,400
DR3v2 NM_003790.2  CGCGGCCCGT GACACGGCGC CCACTTGCCA CCTAGGCGCT CTGGTGGCCC TTGCAGAAGC CCTAAGTACG 1400
          DR3v2Δ6  CGCGGCCCGT GACACGGCGC CCACTTGCCA CCTAGGCGCT CTGGTGGCCC TTGCAGAAGC CCTAAGTACG 1298

                                          1,420                                    1,440                                    1,460
DR3v2 NM_003790.2  GTTACTTATG CGTGTAGACA TTTTATGTCA CTTATTAAGC CGCTGGCACG GCCCTGCGTA GCAGCACCAG 1470
          DR3v2Δ6  GTTACTTATG CGTGTAGACA TTTTATGTCA CTTATTAAGC CGCTGGCACG GCCCTGCGTA GCAGCACCAG 1368

                               1,480                    1,500                     1,520                     1,540
DR3v2 NM_003790.2  CCGGCCCCAC CCCTGCTCGC CCCTATCGCT CCAGCCAAGG CGAAGAAGCA CGAACGAATG TCGAGAGGGG 1540
          DR3v2Δ6  CCGGCTCCAC CCCTGCTCGC CCCTATCGCT CCAGCCAAGG CGAAGAAGCA CGAAC----- ---------- 1423

                                          1,560                                    1,580                                    1,600
DR3v2 NM_003790.2  GTGAAGACAT TTCTCAACTT CTCGGCCGGA GTTTGGCTGA GATCGCGGTA TTAAATCTGT GAAAGAAAAC 1610
          DR3v2Δ6  ---------- ---------- ---------- ---------- ---------- ---------- ---------- 1423

                               1,620
DR3v2 NM_003790.2  AAAACAAAAC AAAAAAAAAA AAAAAAAA 1638
          DR3v2Δ6  ---------- ---------- -------- 1423
```
